# Effects of a multimedia campaign to increase human papillomavirus vaccine acceptance in Dhaka, Bangladesh

**DOI:** 10.1080/21645515.2024.2447105

**Published:** 2025-01-08

**Authors:** Sohail Agha, Sarah Francis, Drew Bernard, Aslam Fareed, Kasrina Azad, Firdausi Qadri

**Affiliations:** a Epidemiology, University of Washington, Seattle, WA, USA; b Research, Behavioral Insights Lab, Seattle, WA, USA; c Research, Independent Consultant, Karachi, Pakistan; d Institute for Developing Science and Health Initiatives (ideSHi), Dhaka, Bangladesh

**Keywords:** Adolescents, HPV vaccination, motivation, ability, multimedia, advertising, campaigns, fogg behavioral model, behavior change interventions, Bangladesh

## Abstract

Increasing the uptake of Human Papillomavirus (HPV) vaccine among adolescent girls is a high priority for the government of Bangladesh. This study examines correlates of HPV vaccine adoption in Dhaka Division, the largest division in Bangladesh. The 18-day vaccination campaign was accompanied by multimedia messages. We use the Fogg Behavior Model (FBM) as the theoretical framework for our analysis. Using a survey instrument based on the FBM, we analyze cross-sectional data collected from 611 caregivers of girls aged 9–17 in Dhaka. Survey data was collected in November and December 2023. Caregivers were recruited via Facebook and Instagram ads and interviewed through the Facebook Messenger App. About one-third of caregivers reported that their child had been vaccinated. We conducted bivariate and multivariate analyses to assess the relationships between the caregivers’ motivation, ability, exposure to the campaign and their child’s vaccination status. Adjusted odds ratios from logistic regression analyses, suggest that caregivers’ motivation, ability, and exposure to the multimedia campaign contributed to vaccine uptake. Caregivers’ motivation to get their child vaccinated was high (74%) but their ability was low (20%). Exposure to campaign messages had a dose-response relationship with vaccine adoption. However, 48% of caregivers remained unexposed to the multimedia campaign. We discuss challenges that the government’s HPV vaccination program is likely to face and identify program-related research questions that are important to answer for the success of future vaccination efforts.

## Introduction

Cervical cancer ranks as the fourth most prevalent cancer among women globally and the second most common cancer affecting women in Bangladesh.^1^ Approximately 12% of cancer cases among women in Bangladesh are attributed to cervical cancer.^2^ A 2011 study in urban and rural Bangladesh showed a high level of willingness to accept the HPV vaccination among ever-married Bangladeshi women despite low levels of knowledge of risk factors for HPV and a limited understanding of how to prevent it.^3^ Awareness of the HPV vaccine was particularly low in rural areas. Studies in Nepal^4^ and India^5^ show similar patterns.

A study in urban Bangladesh also showed high levels of willingness among parents to vaccinate their child even though they had limited knowledge of HPV.^2^ Moreover, parents’ willingness to get their daughter vaccinated was not associated with socio-demographic factors. Data from a national survey, also found no association between vaccine acceptability and socio-economic factors.^3^

In 2016, an assessment of the feasibility of including the HPV vaccine in Bangladesh’s national immunization program was carried out with 30,000 high school girls in Gazipur district in Dhaka Division. Adolescent girls were given two doses of HPV vaccine 6 months apart. Vaccination took place through EPI fixed sites, outreach, and school vaccination sessions. A WHO post-introduction evaluation found that the intervention was well-implemented and achieved a high coverage rate (94%).^6^ The positive outcome from this demonstration project indicated that it was feasible to include the vaccine in the national immunization program. The Gazipur study contributed to the Bangladesh government’s decision to introduce the HPV vaccine nationwide, in partnership with the World Health Organization (WHO), Gavi, the Vaccine Alliance, and UNICEF.^7,8^

In October 2023, over an 18-day period, the HPV vaccine was introduced through the public health system in Dhaka Division. Eligible girls received the HPV vaccine at their educational institutions or designated vaccination centers after registering on the “Vaxpi” app or website. Vaccine introduction was accompanied by a public health awareness campaign highlighting the importance of HPV vaccination to avoid cervical cancer. The campaign included educational programs in schools and community outreach initiatives to inform caregivers about the benefits of vaccinating their daughters against HPV. About 2.3 million vaccines were provided by Gavi for Dhaka Division, which includes the capital and surrounding rural areas.^8^

The government’s awareness campaign aimed to reach a broad audience. Social media and online platforms were used for registration of adolescents as well as information dissemination. Television and radio advertisements, as well as community orientation sessions were conducted for teachers, parents, and religious leaders. This multimedia approach was part of a comprehensive strategy to ensure widespread awareness and acceptance of the vaccine, with a particular emphasis on in-school and out-of-school girls aged 10–14.^8^

Studies have shown that health promotion using digital advertising can increase vaccine information seeking behavior among target audiences.^9^ Studies have also shown that television and radio advertising of public health messages can have an impact on vaccine uptake: a recent, well-designed, US study demonstrated the impact of a multimedia campaign on COVID-19 vaccine uptake;^10^ a recent quasi-experimental study showed that a social media campaign increased COVID-19 vaccine uptake in Nigeria.^11^

No previous study, other than the demonstration project in Gazipur district 2016, has examined drivers of HPV vaccine adoption in Bangladesh. This is the first study to examine the effects of a multimedia campaign to increase HPV vaccination at scale in Bangladesh. Moreover, to the best of our knowledge, no previous study has used a behavioral framework to identify drvers of HPV vaccine adoption in Bangladesh. Given the recency of its introduction and the ambitions of the government to significantly increase HPV vaccine adoption, behavioral insights related to the adoption process may be extremely useful for program designers and implementers in Bangladesh.

## Methods

### Study design

In October 2023, the HPV vaccine was introduced through the public health system in Dhaka Division. In November and December 2023, the Behavioral Insights Lab (BiL) conducted a cross-sectional survey of male and female caregivers of adolescent girls in Bangladesh. The survey was conducted across all divisions of Bangladesh, including the Dhaka Division. For this study, we restrict our analysis to data collected from Dhaka division. We use a posttest only study design to assess campaign effects.

The survey will serve as baseline for the remaining divisions of Bangladesh where the introduction of the HPV vaccine is planned for September 2024.

### Study context

Dhaka City, the capital of Dhaka Division and of Bangladesh, is an economically and culturally vibrant metropolis. As one of the most densely populated areas in the world, the Greater Dhaka Metropolitan Area (GDMA) has a population estimated at 24 million people. It hosts a variety of industries, including textiles and garments, finance, information technology, and manufacturing. It is a major center for international business and investment in South Asia. Dhaka is undergoing rapid infrastructure development, including metro rail systems, flyovers, and urban housing projects. While efforts are being made to modernize Dhaka, major challenges remain in terms of urban poverty. Outside of the GDMA, Dhaka Division encompasses a mix of rural areas, small towns, and emerging urban centers.

Mobile phone ownership is almost 100% in Dhaka division, and smart phone ownership – at 69%- is the highest among all divisions of the country. The Bureau of Statistics reports that 84% of individuals in Dhaka Division use the internet at least once a day.^12^ In early 2024, Facebook was the most widely used social media platform in Bangladesh, with approximately 53 million users, compared with 38 million Tik Tok users and 34 million YouTube users.^13^

### Sampling strategy

We used a multi-stage sampling strategy to collect the data. The sample was split into 14 distinct strata based on seven divisions and gender (male and female). The recruitment strategy involved running 120 separate ad sets tailored to these strata using Meta’s digital advertising platforms (Facebook and Instagram).

### Participant recruitment and data collection

This study builds upon the tremendous, recent, growth in the use of digital research tools. Recruitment was conducted through targeted advertisements on Facebook and Instagram using Virtual Lab, an open-source tool.^14^ The ads were configured to reach potential participants across the study’s multiple strata. The Meta Messenger Chatbot service was employed for survey administration, with an incentive of 100 Bangladeshi Taka (approximately USD 0.85) in mobile credit for survey completion. The advertisements reached 4,318,839 individuals, resulting in 146,032 link clicks. Of those who clicked, 5,279 individuals initiated the survey, and 2,926 respondents provided complete information on nearly all the survey questions. 611 of these respondents were from Dhaka Division.

### Outcome measure

The primary outcome measured was the caregiver’s response to the question “Has a girl-child in your care received an HPV vaccination?” If a caregiver responded in the affirmative, the child was considered to be vaccinated against HPV.

### Study limitations

This study acknowledges several limitations inherent to its design and data collection approach:

1. **Selection Bias**: Although internet use is high in Dhaka, recruitment through social media platforms is likely to exclude caregivers without access to the internet or to social media accounts. As a result, the sample may over-represent digitally connected, younger, urban caregivers, potentially limiting the generalizability of findings to the broader caregiver population in Bangladesh.
2. **Self-Reported Data**: The reliance on self-reported vaccination status introduces the potential for recall or social desirability bias. Respondents may have over-reported or under-reported HPV vaccine uptake due to memory inaccuracies or perceived expectations regarding vaccine uptake.
3. **Single Time Point Data Collection**: Cross-sectional survey data provides only a snapshot of caregiver attitudes, beliefs, and behaviors, precluding causal inferences. A longitudinal design is more likely to capture temporal changes and provide insights into how motivations, abilities, and vaccination behaviors evolve over time.

### Sample size determination

One consideration in determining the sample size for the survey was to have a higher number of respondents from the two largest divisions of Bangladesh, Dhaka Division (estimated population of 44 million) and Chittagong Division (estimated population of 33 million). At the same time, it was important to have a sufficient number of respondents from other divisions to enable a comparison of changes in the vaccination rate over time.

We are not aware of any estimate of the HPV vaccination rate among adolescent Bangladeshi girls prior to this survey. The sample size was calculated conservatively, based on an assumed 50% prevalence of HPV vaccination. We assumed a design effect of 1.5 for our sample size calculations. Our calculation indicated that a sample of 600 would be sufficient to ensure a margin of error of six percentage points for the outcome, HPV vaccination.

A total sample size of 3,000 caregivers was determined for all divisions in the country, with a margin of error of 2.7 percentage points. The survey was designed to collect data from 600 respondents from Dhaka Division, 600 from Chittagong and 300 from each of the remaining divisions. As mentioned earlier, the final sample size for Dhaka Division was 611 caregivers of adolescent girls.

### Selection and operationalization of variables

Our study used the Fogg Behavior Model (FBM) to select variables for the analysis. The FBM states that behavior happens when motivation, ability, and a prompt happen in the same moment.^15^ We collected data on motivation and ability because of their significant impact on vaccine uptake behaviors, as demonstrated in other studies.^16,17^

We created binary variables for motivation and ability. To measure motivation, we asked participants to respond to the statement, “Getting the girl who is in my care vaccinated against HPV is important to me.” Those strongly agreeing with this statement were classified as having a high level of motivation. To measure ability, participants were asked to respond to the statement, “Getting the girl vaccinated against HPV is difficult,” and respondents who strongly disagreed with this statement were classified as having high ability. Our methodology is identical to the approach used in previous research measuring these two constructs^18^ and builds upon the established correlations between motivation, ability and vaccine uptake.^16^

To measure the prompt to behavior, we asked respondents “Have you ever seen or heard any advertisements or messages about the HPV vaccine in the last 3 months?” Caregivers who responded in the affirmative were asked “How often have you seen advertisements or heard messages about HPV vaccine?” A three-category exposure variable was created based on these two questions, which measured the frequency of advertising exposure reported by caregivers: never, 1–3 times, more than 3 times.

We measured caregiver’s age, gender, level of education, and residence in urban or rural areas. These were used as control variables in the multivariate analysis.

### Data analysis

Univariate analysis examined frequency distributions of variables that might be associated with HPV vaccine uptake, including motivation, ability, and exposure to HPV messaging/advertising. Frequency distributions were also examined of all control variables.

Bivariate analysis explored the relationships between motivation, ability, advertising exposure, and the outcome variable – the girl child’s HPV vaccination status. We also examined the relationships between control variables and HPV vaccination status.

Multivariate logistic regression analysis was conducted to identify factors associated with HPV vaccination. The STATA statistical package was used for the analysis. Chi-squared tests of independence were conducted at the bivariate level. Adjusted odds ratios from logistic regression analyses were estimated at the multivariate level.^19^ Associations between variables were considered statistically significant at *p* < .05.

## Results

Table 1, Column 1, shows frequency distributions of caregivers’ sociodemographic characteristics, motivation, ability, exposure to HPV vaccine advertising and the outcome, HPV vaccination status. About 34% of respondents were 18–29, 44% were 30–39 and 22% were ages 40 and older. Nearly 51% of caregivers in our sample were women, 46% were men, and the remaining 3% did not report their gender or reported their gender as non-binary. The sample was relatively well-educated, with 46% of respondents having a higher secondary or bachelor’s education and 37% having a master’s education. A majority of respondents were from urban Dhaka (56%).

**Table 1.** Frequency distributions of caregiver characteristics and cross-tabulations between characteristics and caregiver reports that their female child had been vaccinated.

	(1) Sample Characteristics (*n* = 611)	(2) % of Caregivers Who Report That Their Female Child Has Been Vaccinated (*n* = 611)	(3) *p*-Value
**Age**			
18–29	34.0	28.4%	.047
30–39	43.5	31.6%	
40+	22.4	40.9%	
**Gender of Caregiver**			
Man	46.3%	30.4%	.455
Woman	51.2%	34.8%	
Not reported/non-binary	2.5%	26.7%	
**Education**			
Less than higher secondary	16.7	26.5%	.279
Higher secondary or bachelors	46.3	32.5%	
Masters	37.0	35.4%	
**Residence**			
Town/Rural	43.9%	29.1%	.106
City	56.1%	35.3%	
**Motivation** (Getting the girl in my care vaccinated against HPV is important)			
Low	26.4%	19.3%	<.001
High	73.6%	37.3%	
**Ability** (Getting the girl child vaccinated against HPV is easy)			
Low	79.7%	26.7%	<.001
High	20.3%	55.6%	
**Frequency of having seen/heard messages about the HPV vaccine**			
Never	47.8%	13.7%	<.001
1–3 times	28.1%	40.1%	
More than 3 times	24.1%	61.2%	
**Has a girl child in your care received an HPV vaccine?**			
No	67.4%	-	
Yes	32.6%	-	
Total	100%	**32.6%**	

The level of motivation to get their child vaccinated was quite high among caregivers: 74% of caregivers strongly agreed that getting the girl in their care vaccinated against HPV was very important to them. By contrast, caregivers’ ability to get their child vaccinated was low: only 20% of caregivers strongly disagreed that getting the child in their care vaccinated against HPV was difficult. In terms of exposure to the government’s HPV campaign, 48% of caregivers had not seen or heard a message related to the HPV vaccine, 28% had been exposed to HPV vaccine messages 1–3 times, and 24% had been exposed to HPV vaccine messages more than 3 times. Overall, 33% of caregivers reported that their girl child had been vaccinated against HPV.

Table 1, Column 2, shows the relationship between caregiver characteristics, motivation, ability, exposure to HPV messaging, and caregiver reports that their girl child had received the HPV vaccination. The HPV vaccination rate increased with the caregiver’s age: 28% of caregivers 18–29, 32% of caregivers 30–39, and 41% of caregivers 40 and older reported that their child had been vaccinated. There was no significant difference in the HPV vaccination rate by caregiver’s gender, education, or residence in urban versus rural Dhaka Division.

We found strong bivariate relationships between a caregiver’s motivation, ability and whether their child received the HPV vaccination: there was an 18-percentage point difference in HPV vaccination between caregivers with high motivation and caregivers with low motivation (37% versus 19%); there was a 29-percentage point difference in HPV vaccination between caregivers with high ability and caregivers with low ability (56% versus 27%). The relationship between advertising exposure and vaccine uptake was also strong: the vaccination rate was 14% among caregivers who had not seen messages related to the HPV vaccine, 40% among caregivers who had seen or heard 1–3 messages, and 61% among caregivers who had seen or heard 3 or more messages.

Table 2, Model 1, shows the odds ratio of a girl child in Dhaka Division receiving an HPV vaccination after adjusting for the socio-demographic characteristics, motivation, and ability of caregivers. After adjusting for other variables in the model, the caregiver’s age was no longer associated with vaccine uptake. Consistent with our findings at the bivariate level, gender, education, and urban residence were also not associated with HPV vaccination. Caregivers’ motivation and ability were strongly associated with HPV vaccination. Specifically, caregivers with high motivation had a 1.92 higher odds ratio (*p* = .005), while caregivers with high ability had a 2.92 higher odds ratio of vaccinating their child (*p* < .001).

**Table 2.** Adjusted odds of a female child in Dhaka being vaccinated against HPV (caregiver reports).

	Model 1 Odds Ratio (*n* = 611)	*p*-Value	Model 2 Odds Ratio (*n* = 611)	*p*-Value
**Age**				
18–29	1.00		1.00	
30–39	1.14 (0.75–1.73)	.530	0.97 (0.62–1.52)	.908
40+	1.41 (0.85–2.32)	.184	1.36 (0.79–2.34)	.262
**Gender of Caregiver**				
Man	1.00		1.00	
Woman	1.27 (0.74–2.13)	.203	1.10 (0.74–1.64)	.620
Not reported/non-binary	0.89 (0.27–2.97)	.848	1.03 (0.29–3.71)	.961
**Education**				
Less than higher secondary	1.00		1.00	
Higher secondary or bachelors	1.26 (1.76–3.00)	.393	1.10 (0.63–1.94)	.729
Masters	1.07 (0.61–1.88)	.807	0.96 (0.53–1.75)	.893
**Residence**				
Town/Rural	1.00		1.00	
City	1.23 (0.85–1.78)	.267	1.09 (0.73–1.61)	.678
**Motivation**				
Low	1.00		1.00	
High	1.92 (1.22–3.02)	.005	1.48 (0.91–2.41)	.111
**Ability**				
Low	1.00		1.00	
High	2.92 (1.90–4.49)	<.001	2.12 (1.32–3.39)	.002
**Frequency of having seen/heard messages about the HPV vaccine**				
Never			1.00	
1–3 times			4.02 (2.53–6.40)	<.001
More than 3 times			7.64 (4.66–12.51)	<.001
Pseudo R-squared	6.65%		16.74%	

Table 2, Model 2, shows the adjusted odds of a girl child in Dhaka Division receiving an HPV vaccination after adjusting for socio-demographic characteristics, motivation, ability, and exposure to HPV messaging. Model 2 shows that exposure to advertising had a dose-response relationship with vaccine adoption: caregivers exposed to 1–3 advertisements had a 4.02 times higher odds ratio of vaccine adoption than those not exposed to advertising (*p* < .001); caretivers exposed to more than three advertisements had a 7.64 times higher odds ratio of vaccinating their child compared to those with no exposure (*p* < .001). After adjusting for exposure to advertising in Model 2, a caregiver’s ability to get their child vaccinated remained a significant correlate of vaccine uptake: the odds ratio of a girl child being vaccinated against HPV remained twice as high if her caregiver had high ability to get her vaccinated (aOR = 2.12). However, the correlation between a caregiver’s motivation and vaccine uptake became non-significant after adjusting for exposure to HPV vaccine messaging. This finding suggests that one of the mechanisms through which HPV vaccine messaging contributed to vaccine uptake in Dhaka Division may have been by increasing a caregiver’s motivation to get their child vaccinated. It is noteworthy that advertising exposure had a strong, independent, effect on HPV vaccination.

Overall, Model 2 explained approximately 17% of the variance in the outcome, as indicated by the Pseudo R-squared value. The goodness of fit test showed a Pearson chi-squared (225) value of 248.10 with a *p*-value of 0.1369. The area under the ROC curve was 0.77, which is considered acceptable.

**Figure 1.**
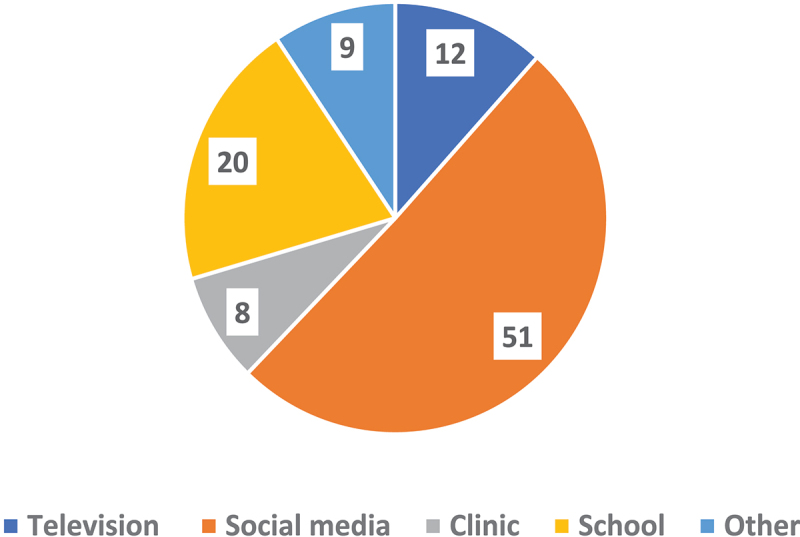
Source of message among caregivers exposed to HPV advertising (*n* = 319).

Figure 1 shows the source of HPV messages among those exposed to advertising. Of the 319 caregivers (52% of the sample) who reported being exposed to messages related to the HPV vaccine, 51% cited social media as the source of the message, 20% reported schools, 12% reported television, and 8% reported a clinic as being the source.

## Discussion

This study provides valuable insights about how motivation, ability, and exposure to vaccine advertising influenced HPV vaccine adoption in Dhaka, the largest administrative division in Bangladesh. The study is among the first to apply the Fogg Behavior Model (FBM) to analyze vaccine adoption in a low-and middle-income country (LMIC). It uniquely demonstrates the power of multimedia campaigns to influence HPV vaccine adoption in resource-constrained settings, providing a replicable approach for other LMICs interested in introducing HPV vaccines in their national immunization programs.

Furthermore, the innovative use of digital tools for data collection offers a cost-efficient, time-saving approach, showcasing how public health campaigns in LMICs can adapt to technological advancements to generate actionable insights. Combining the use of a practical behavior model awith inexpensive, digital, data collection has the potential to enhance current understandings of the process through which public health interventions work in diverse socio-cultural contexts. The need for practical behavior change frameworks to help implementers and researchers use behavioral science in low-resource settings has been highlighted elsewhere.^16^

This study employs a cross-sectional design, capturing a snapshot of caregiver motivation, ability, and vaccine uptake following the implementation of a multimedia campaign supported by the government and its partners. This is not an experimental study, and no causal inferences have been drawn from its findings. While the study design limits our ability to establish causation, the study does provide valuable insights about the influence of the campaign. To enhance the robustness of the findings, we employed multivariate logistic regression to control for a range of variables. Adjusting for these variables increases the validity of our inferences, ensuring that the observed relationships between motivation, ability, and vaccine uptake are not merely artifacts of underlying sociodemographic differences. This statistical rigor supports the strength of the conclusions despite the inherent limitations of a cross-sectional study design.

The association between motivation and vaccine uptake is consistent with expectations of the Fogg Behavior Model (FBM), which posits that the presence of motivation, ability, and a prompt in the same moment leads to a behavior. Our study finds that a caregiver’s motivation has a strong association with vaccine adoption by their child. We found that a caregiver’s motivation to have their girl child vaccinated was high following the HPV vaccine’s introduction in Dhaka and the multimedia campaign that was implemented to promote it. By November/December 2023, 74% of caregivers in Dhaka Division reported that they considered it very important for their girl child to get vaccinated.

At the same time, it is noteworthy that about half the caregivers in Dhaka Division were not exposed to the multimedia campaign, which included both mass media, social media, and community-level awareness raising. Determining the characteristics of caregivers who remain unexposed to the government’s campaign is an extremely important research and programmatic priority. What communication channels do caregivers who were not exposed to the multimedia campaign have access to or pay attention to? What strategies can be designed to reach these caregivers? Is it possible that they were exposed to campaign messages but could not recall the messages because the messages did not resonate with them? Since the HPV campaign’s messaging was tied closely to cancer prevention, it may be worthwhile to explore other messages that may resonate more with caregivers in Bangladesh.

The finding that a caregiver’s ability is a significant predictor of HPV vaccination after adjusting for socio-demographic variables, suggests that ability factors remain a major challenge for HPV vaccine uptake in Dhaka Division. Even after the government made the HPV vaccine widely available in Dhaka Division, the level of ability of caregivers remained low: only 20% of caregivers strongly disagreed with the statement that it was difficult to get the girl in their care vaccinated against HPV. These findings highlight the importance of identifying the specific ability barriers faced by caregivers in Dhaka. The FBM considers ability barriers to include not having sufficient time, not having enough money, having to make a physical effort to adopt a behavior, having to make a mental effort to adopt a behavior and the behavior not fitting into a person’s routine. Research is needed to determine which of these ability barriers are most important in Dhaka Division and what can be done to overcome them. Once identified, regular assessments of whether there is a reduction in the most important ability barriers will help inform policymakers and implementers on what additional interventions are needed to enhance caregivers’ capacity to facilitate vaccine uptake by their children.

This study contributes novel insights by quantifying the impact of advertising exposure, identifying it as a crucial prompt for vaccine adoption in LMICs. While the campaign had a powerful association with the adoption of the HPV vaccine, our findings suggest that a lot of work remains to be done to get the vaccination rate closer to the government’s target of 90%. Our survey, conducted immediately after the government’s 18-day push to get the vaccine out through the public health system, showed that 33% of adolescent girls had been vaccinated.

### Limitations

The data collection approach taken in this study is both pragmatic and cost-efficient. However, it has several important limitations. The first is the use of a cross-sectional survey. Since predictor variables and HPV vaccination were measured at the same time, no causal inferences can be drawn. The second is that the study is based on a non-representative sample. This limits the generalizability of the study findings. The third is that the outcome relies on reported vaccination behavior.

### Strengths

A strength of our study is that it is based on a behavior model that has been tested across multiple health behaviors in LMICs, including in India and Pakistan.^15^ Another strength of the study is that it adjusts for sociodemographic differences between caregivers before reaching conclusions about the relationships between variables.

The recruitment and data collection strategy used in this study is much faster and much less expensive than that used in the standard representative household surveys. Conducting a household survey is a resource- and time-intensive exercise. In our case, this would have meant finding and hiring a survey research organization with expertise in conducting household surveys in Bangladesh. The cost and time required to administer a representative household survey of Dhaka Division would have been far beyond the budget that was available to us. In addition, the planning and operations required to conduct a household survey, which includes listing households, hiring teams to conduct household interviews, training interviewers, monitoring data collection and conducting face-to-face interviews, would have taken several months. By contrast, the digital survey data collection was completed in November and December 2023, immediately following the HPV vaccine introduction in Dhaka Division. Following the study’s IRB approval on October 28th, we were able to pretest the survey instrument and complete the survey data collection in less than two months – by December 21st.

The cost and time required to conduct a household survey is often prohibitive for field-based programs in LMICs. This often translates into very limited programmatic research being conducted in countries with have limited resources for research. This study shows that, by conducting a rapid, inexpensive survey, it is possible to provide insights to help program designers better understand the role of multiple factors that influence vaccine uptake – and could inhibit the effectiveness of vaccine rollouts.

The global interest in behavioral insights related to vaccine adoption is relatively recent. This COVID-19 crisis highlighted the need for innovative approaches to address vaccine hesitancy.^20,21^ A handful of recent studies in Bangladesh have used a behavioral framework to examine factors associated with the adoption of the COVID-19 vaccine.^22–25^ Yet, only one study has applied a behavioral framework to assess parents’ intentions to vaccinate their child against HPV.^26^ This study is based on data collected prior to Bangladesh’s introduction of the single dose HPV vaccine.

In addition to being the first study to examine HPV vaccine adoption following its introduction in Dhaka Division, our study advances the field by employing the Fogg Behavior Model (FBM), a novel and practical framework suited to use in a low resource context. Previous studies have demonstrated its explanatory power for multiple health behaviors, including COVID-19 vaccination, condom use, and iron folate supplementation in LMICs such as Nigeria, Pakistan, and India. By focusing on the interplay of motivation, ability, and prompts, the FBM provides actionable insights for program implements and designers to develop vaccination interventions using behavior science.

## Conclusions

Our study reflects the dynamics of HPV vaccine adoption in a major population center in Bangladesh. The findings highlight the importance of caregiver motivation and ability in driving HPV vaccine adoption. The study shows that a multimedia campaign may contribute to increasing the HPV vaccination rate by increasing caregivers’ exposure to vaccine messaging. The findings also suggest that increasing caregiver motivation may be one of the mechanisms through which advertising contributes to vaccine adoption.

While achieving a 33% HPV vaccination rate following an 18-day vaccine introduction is impressive, it indicates the need for continued efforts to achieve the target of 90% HPV vaccination among girls 10–14.

Digital surveys can be a rapid, inexpensive, tool for programs to generate insights about the impact of multimedia campaigns. Particularly if they use a behavior model, surveys can provide an understanding of whether a campaign is progressing as per expectation, and whether expected relationships between variables such as motivation, ability and behavior are observed. In addition, they can indicate whether awareness campaigns serve as a prompt to vaccine adoption.

## Data Availability

Data will be made available upon reasonable request to the first author.
